# Supplementary material for: Impact of Body Mass Index on Outcomes in Pediatric Allogeneic Hematopoietic Stem Cell Transplantation Recipients: A Single-Center Retrospective Study
Source: Nutrients. 2024 Oct 25;16(21):3638. doi: 10.3390/nu16213638 (PMC11547439; doi:10.3390/nu16213638)
Supplement: Supplementary file 1 [file nutrients-16-03638-s001.zip › nutrients-3270108-supplementary.pdf]

Original Article

Impact of Body Mass Index on Outcomes in Pediatric Allogeneic Hematopoietic Stem Cell Transplantation Recipients: a Single-Center Retrospective Study.

Stefania Braidotti [1†], Debora Curci [2†], Davide Zanon [3], Alessandra Maestro [3], Antonella Longo [1], Nicole De Vita [1], Natalia Maximova [1*]

Supplementary Results:

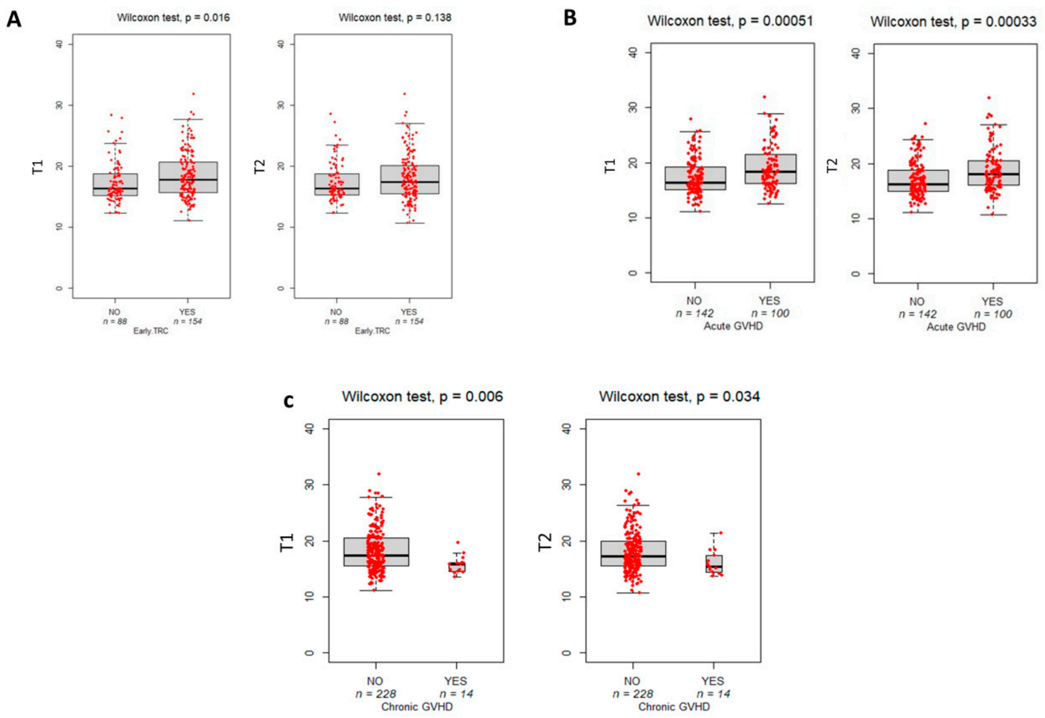

Figure S1. Correlation between BMI and early and late transplant complications. Boxplots Boxplot comparing BMI levels assessed before (T1) and after first-line treatment (T2) and the risk of early TRC (Panel A), acute GVHD (Panel B), and chronic GVHD (Panel C). The bold horizontal line represents the median value. P-values are from the Wilcoxon test. GVHD: graft-versus-host disease.

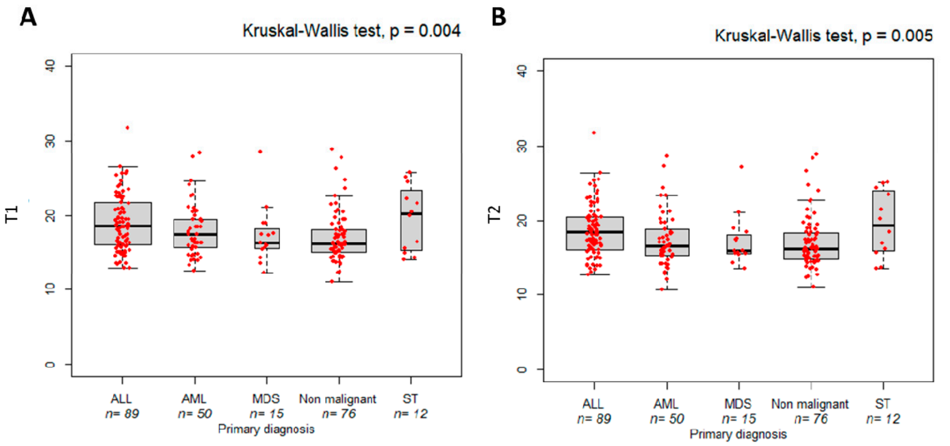

Figure S2. Correlation between BMI and primary disease. Boxplots compare the BMI assessed before (T1, Panel A) and after first-line treatment (T2, Panel B) across five primary disease groups. The bold horizontal line represents the median value. ALL: Acute Lymphoblastic Leukemia, AML: Acute Myeloid Leukemia (AML); MDS: Myelodysplastic syndrome (MDS); HSCT: hematopoietic stem cell transplantation. ST: Solid Tumor.

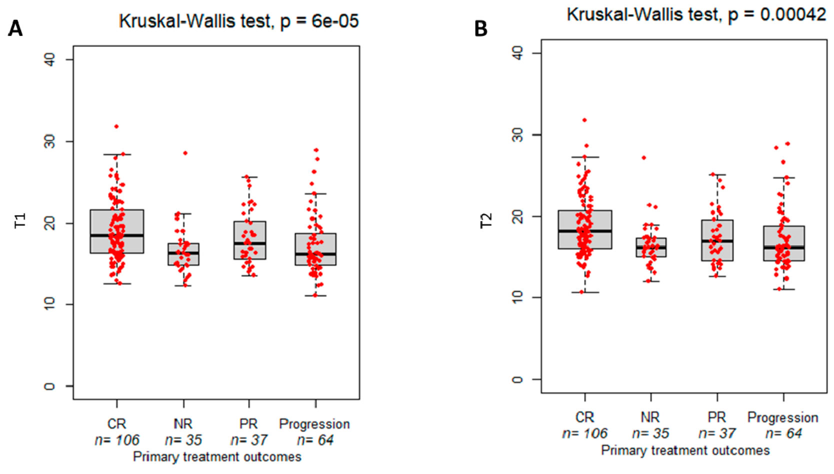

Figure S3. Correlation between BMI and primary treatment outcomes. Boxplots compare the BMI assessed before (T1, Panel A) and after first-line treatment (T2, Panel B) grouped by primary treatment outcomes (CR = complete response; NR = Nonresponse, PR = partial response, and progression). The bold horizontal line represents the median value.

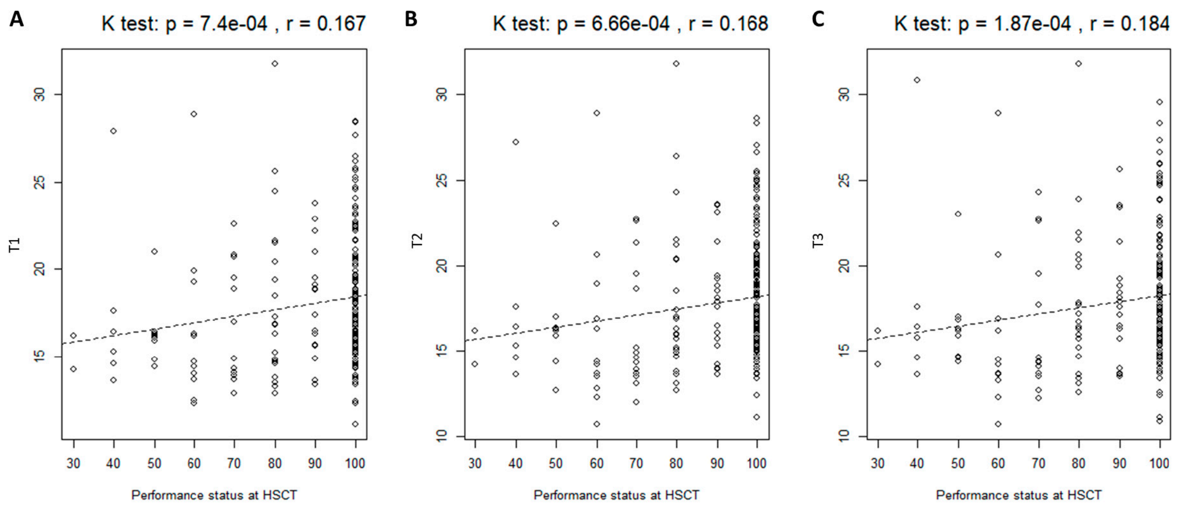

Figure S4. Correlation between BMI at different time points and performance status at HSCT. BMI was assessed before (T1, Panel A), after first-line treatment (T2, Panel B), and before allo-HSCT (T3, Panel C).

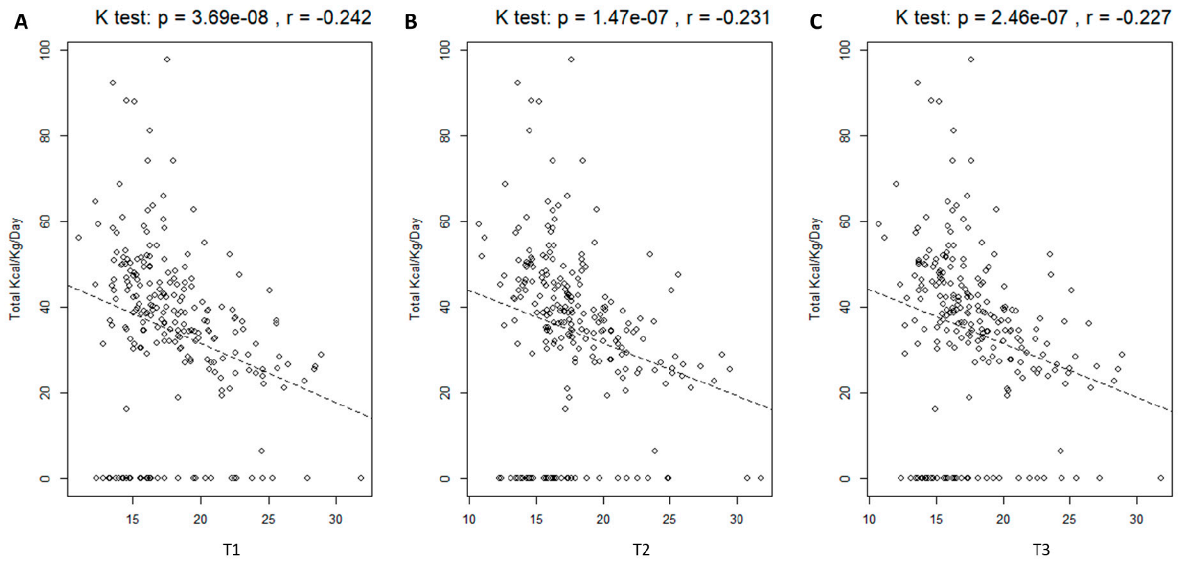

Figure S5. Correlation between BMI at different time points and NPT (Total Kcal/Kg/Day). BMI before (T1, Panel A) and after first-line treatment (T2, Panel B) and before allo-HSCT (T3, Panel C).

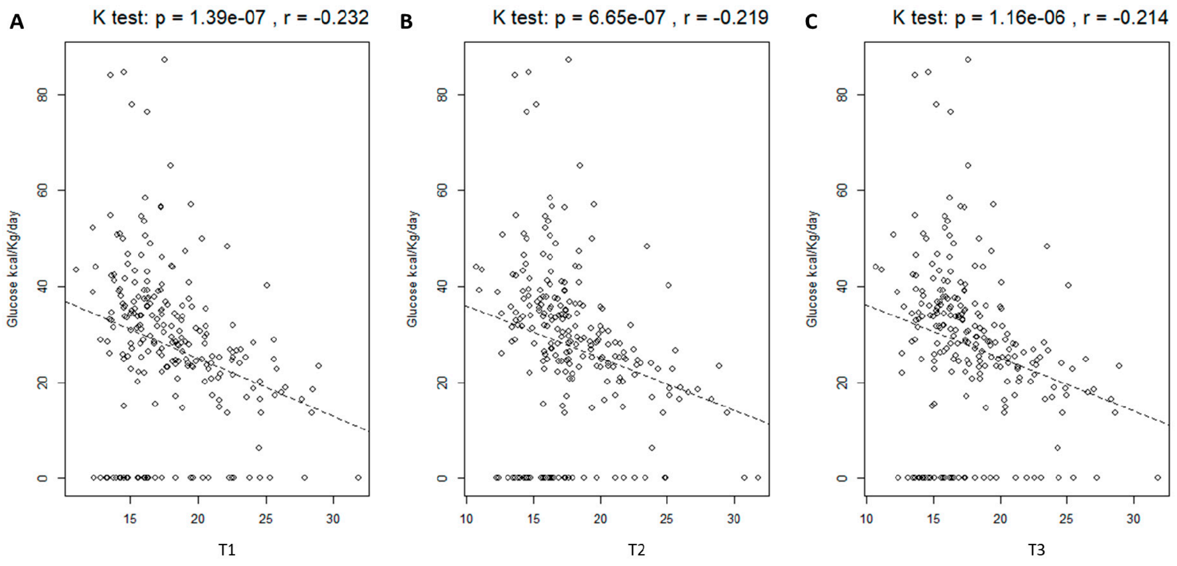

Figure S6. Correlation between BMI at different time points and NPT (Glucose Kcal/Kg/Day). BMI before (T1, Panel A) and after first-line treatment (T2, Panel B) and before allo-HSCT (T3, Panel C).

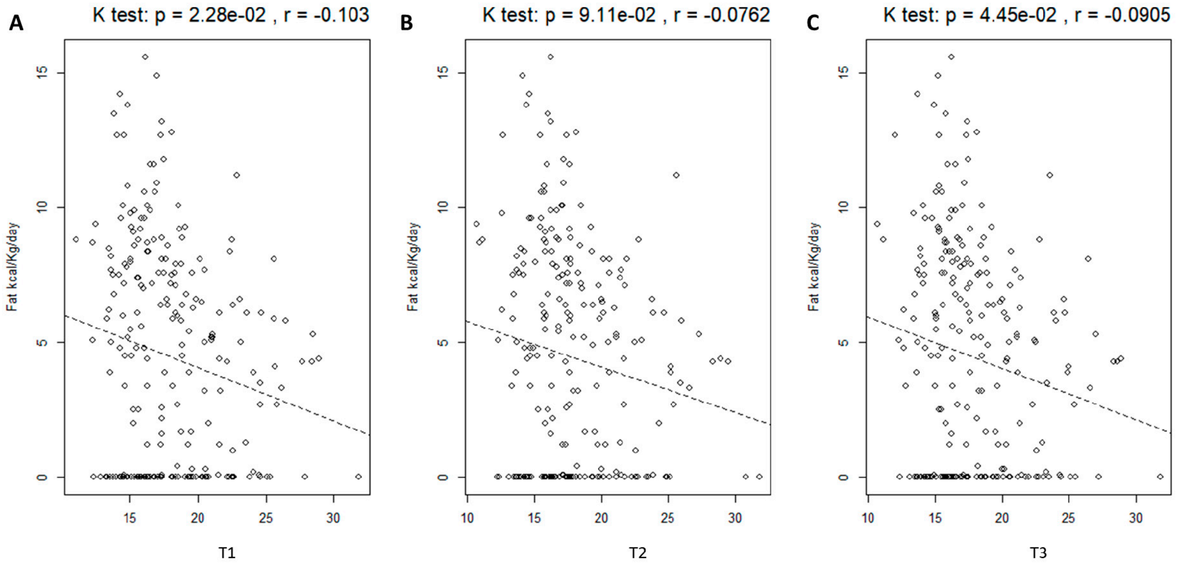

Figure S7. Correlation between BMI at different time points and NPT (Fat Kcal/Kg/Day). BMI before (T1, Panel A) and after first-line treatment (T2, Panel B) and before allo-HSCT (T3, Panel C).

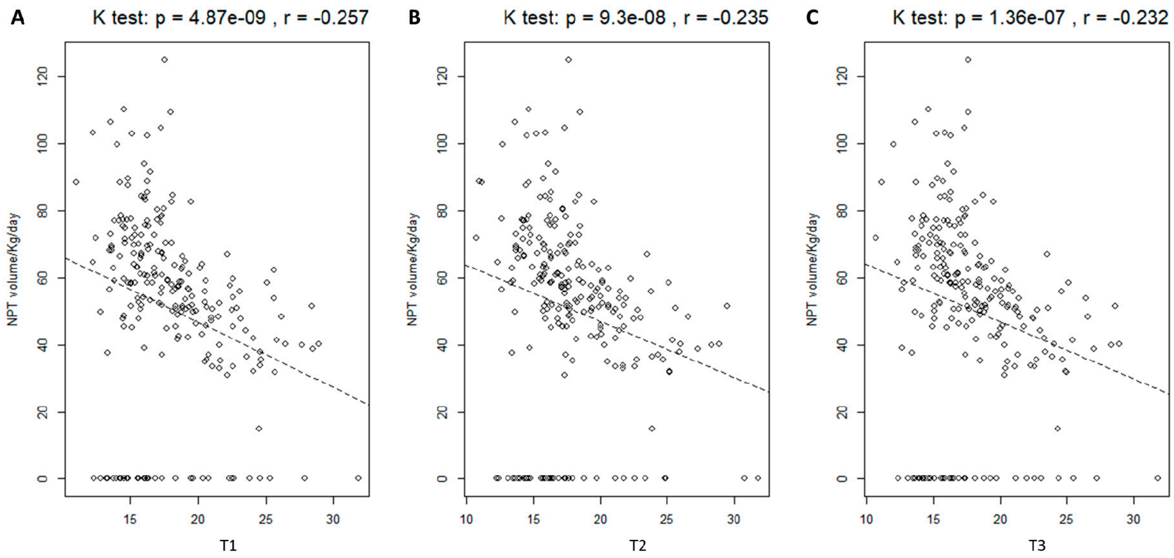

Figure S8. Correlation between BMI at different time points and NPT (volume/Kg/Day). BMI before (T1, Panel A) and after first-line treatment (T2, Panel B) and before allo-HSCT (T3, Panel C).
